# Leadership in sustainment of Individual Placement and Support model: a comparative case study in Finland

**DOI:** 10.1186/s12913-025-12495-1

**Published:** 2025-03-04

**Authors:** Jaakko Harkko, Hilla Nordquist, Anne Kouvonen

**Affiliations:** 1 Faculty of Social Sciences, University of Helsinki, Helsinki, Finland; 2 South-Eastern Finland University of Applied Sciences, Kotka, Finland; 3 Department of Public Health, University of Helsinki, Helsinki, Finland; 4 Centre for Public Health, Queen’S University Belfast, Belfast, Northern Ireland

**Keywords:** Supported employment, Individual Placement and Support, Leadership, External context, Organizational context, Mental health services

## Abstract

**Background:**

Individual Placement and Support is an evidence-based practice designed to assist individuals with psychiatric disorders in gaining employment. Contextual factors such as policies, funding, and organizational structures are widely acknowledged as barriers and facilitators to the sustainment of evidence-based practices. This study focuses on leadership’s role in driving the sustainment process and navigating the dynamic interplay between internal and external contexts following a national development project in Finland.

**Methods:**

We used qualitative data from semi-structured interviews. Participants included outside experts such as trainers and researchers (*n* = 4), leaders from the implementing sites, including medical directors, nursing directors, administrators (*n* = 11), project managers and team leaders (*n* = 6), and frontline workers (*n* = 8). We performed an abductive thematic analysis to identify patterns and themes within the data.

**Results:**

We identified three Leadership Approaches for IPS Model Sustainability that differentiated agencies with more robust sustainment strategies from those with less robust ones. The Visionary Approach included championing recovery-oriented principles to foster organizational readiness and aligning IPS with organizational goals. The Proactive Approach was characterized by early and deliberate planning to minimize challenges, leveraging IPS fidelity evaluations for quality improvement. Leaders employing the Collaborative Approach emphasized effective facilitation within governance structures, including the integration of IPS into the strategies of administrative units.

**Conclusions:**

The study presents three leadership approaches that differentiate implementing agencies with more robust IPS model sustainment strategies from those with less robust ones. It introduces and empirically explores a conceptual model that can be used both in research and practice.

**Supplementary Information:**

The online version contains supplementary material available at 10.1186/s12913-025-12495-1.

## Background

The emphasis on evidence-based practices (EBPs) and their implementation in psychiatric rehabilitation has increased over recent decades. Ideally, high-quality mental health interventions stand out by being well-defined, aligning with client goals and societal objectives, and demonstrating efficacy and effectiveness, among other key qualities [1]. The Individual Placement and Support (IPS) model is an EBP in mental health care that integrates vocational rehabilitation with mental health treatment through a multidisciplinary team approach [2]. Meta-analyses have shown substantial client benefits from this model [3, 4]. Developed in the US in the 1990s, the IPS model has since gained international traction, finding application across numerous countries such as the US, Canada, Australia, and many Western European countries. Although the IPS model has expanded internationally, challenges in its system-wide adoption have been recognized [5].

Recent systematic reviews indicate two less explored research areas that may contribute to this knowledge translation gap. First, in IPS studies, the sustainability of services is an aspect that has received less attention than the implementation of the model [6], a common feature also in general health service delivery studies [7]. The IPS implementation research shows that leadership is crucial in adopting and sustaining the IPS model. We use the term “leadership” as it is defined in implementation science, referring to individuals who directly or indirectly influence the implementation of EBPs at any level of an organization, including executive leaders, middle managers, frontline supervisors, and team leaders [8]. In this study, we focus on leaders at all levels within the boundaries of the implementing agency, as defined in the IPS model guidelines [9]. Leaders facilitate the integration of recovery orientation and the IPS model into organizational strategies [10]. They facilitate organizational changes and integrating services across different professions [11–13]. The capability of leaders to make affirmative decisions, such as reallocating staff positions from non-evidence-based practices [14, 15] or involving steering committees in strategic decision-making for program implementation [12, 14, 16], is also crucial. Facilitative leadership includes building effective relationships and engaging corporate leaders in complex organizational structures (10). Commitment to fidelity standards is also likely to facilitate the successful implementation of the IPS model [17, 18] and the model’s sustainability [13]. These results show the importance of leadership in decision-making, shaping organizational culture, and commitment to quality improvement in the local organizational context.

The second knowledge gap lies in the relatively limited number of studies that examine external context as a critical determinant of sustainability [6, 7]. In this study, we define external context as any condition or circumstance external to the agency implementing the IPS model. We focus on the aspects of the external context that research has identified as important for implementing and sustaining the IPS model, including national and regional strategies, legislation, or funding mechanisms [6]. The IPS implementation research shows that the collaboration between the administrative agencies [12, 19] and the continuity of administrative support for local sites [20] may affect program sustainability. Conversely, non-commitment from external financial authorities following a project’s conclusion may result in discontinuing the service [15]. Also, the research shows that the leaders face practical challenges in planning and collaborating with internal and external partners [15, 21], including those arising from conflicting demands and institutional logic of the collaborating partners [10, 22]. Despite the importance of managing these demands and relationships, this form of collaboration may hinge on informal strategies and cultivating mutual trust among key stakeholders [10] rather than systematic implementation approaches. The model originators have created a dissemination model to foster collaboration between administration representatives and support organizations at the state/regional level in the US [19, 23]. This strategy suggests that the model’s sustainability is likely influenced by the cohesiveness of actions among administrative and agency leaders, emphasizing taking into account the varying influences of both administrative and agency-level actors.

### Leadership, external context, organizational context, and sustainment – a conceptual model

**Fig. 1:**
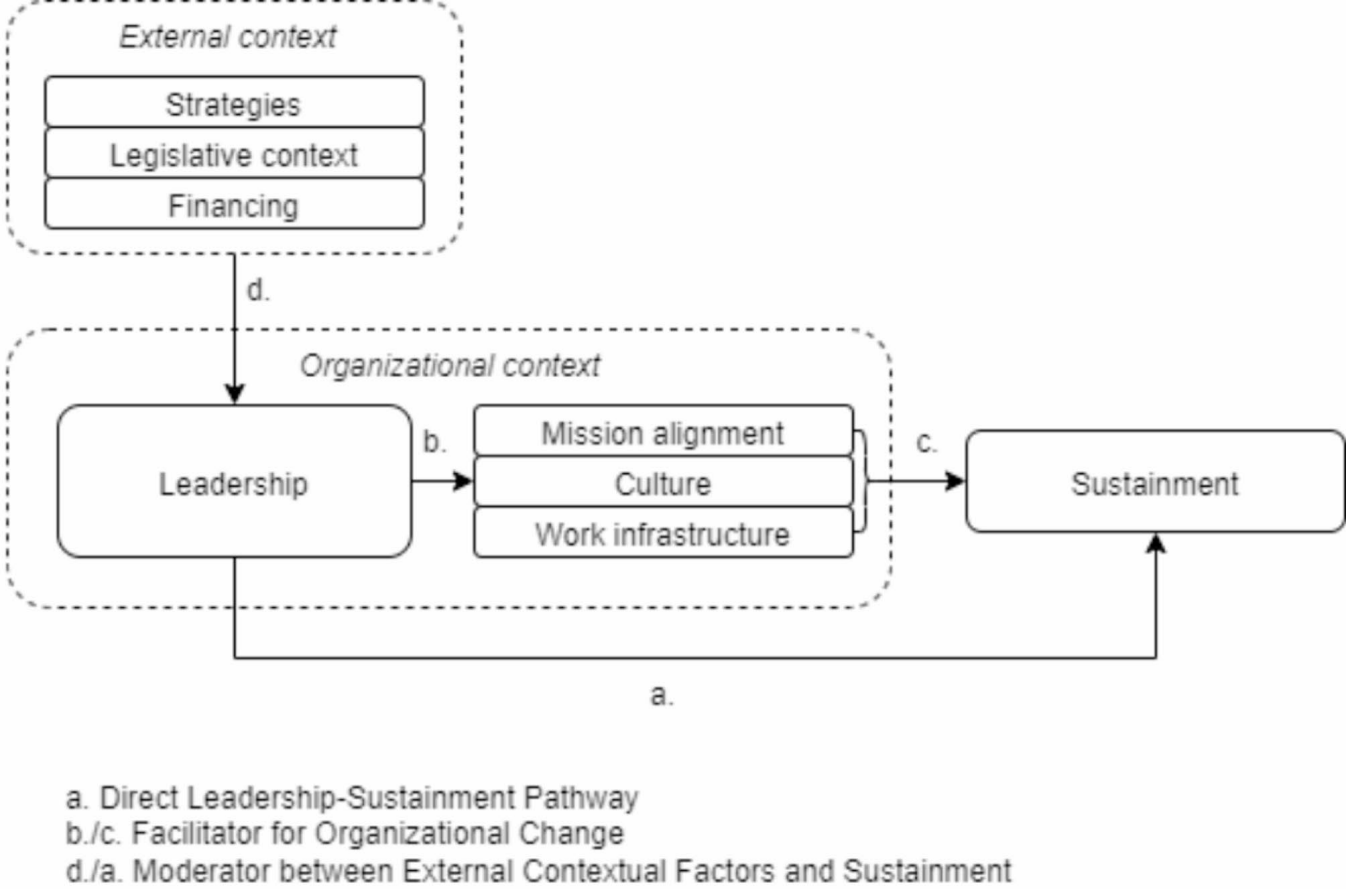
Leadership, external context, organizational context, and sustainment– a conceptual model

This study builds on a conceptual model from a scoping review that highlights leadership as being associated with contextual barriers and facilitators, and the sustainment of the IPS model [6]. The conceptual model, an adapted version of Consolidated Framework for Implementation Research (CFIR) [24], was derived from findings from 59 empirical IPS model implementation studies. The model proposed relational properties among the implementation outcomes and the barriers and facilitators influencing these outcomes, commonly referred to as ‘determinants’ in implementation science. Figure 1 illustrates the elements of this model related to leadership, which were confirmed through data analysis in this study. The figure distinguishes between three potential pathways associated with the leaders’ role in relation to sustainability: ‘Direct Leadership-Sustainment Pathway’, ‘Facilitator for Organizational Change’, and ‘Moderator between External Contextual Factors and Sustainment’. We use the term ‘moderator’ while acknowledging that leaders engage in dynamic interactions with the stakeholders, resulting in highly contingent and inherently uncertain outcomes rather than deterministic [25]. A pathway from external context to sustainment, not moderated by leadership, could also have been acknowledged. Our data did not provide clear insights into this pathway; hence, it is not represented in the conceptual model.

### Aims of the study

The objective of this study is to explore leadership’s role in driving the sustainment process and navigating the dynamic interplay between internal and external contexts in sustaining the IPS model. To do so, we answer the following research questions:

- What leadership characteristics are associated with sustainability of the IPS model?
- How do leaders navigate contextual barriers and facilitators to sustain the IPS model?

## Methods

### Study design

We conducted a qualitative comparative case study using interviews with professionals participating in the implementation process. With this approach, we aim to describe the complexity and distinctiveness of a specific policy process embedded in institutions in a “real-life” environment [26, 27]. The unit of analysis in this study is the leadership’s role in the transition from project implementation to sustained services after a national demonstration project [28]. We compare and contrast the transitions to sustained services among five implementing units, aiming to identify common patterns, themes, and variations in the data.

Regarding the IPS model expansion, the initial Finnish demonstration projects are highlighted as a ‘typical case’ since the model originated in the US and is now utilized in over 20 countries. Regarding the transition from project implementation to sustained services, the case under examination represents a ‘critical case’ due to two concurrent conditions. The inclusion of the IPS model in the National Mental Health Strategy [29] indicated a long-term commitment to disseminating the IPS model. However, no new laws or funding sources were established to facilitate the sustained implementation. These conditions may be considered ideal for exploring leadership’s role in driving sustainment, particularly in identifying policy-level barriers and facilitators that influence leadership.

### Context

Between 2021 and 2023, the Finnish government-funded national multi-site IPS demonstration project, including several regional pilots across the country, was implemented as part of the National Mental Health Strategy [29]. At the time of data collection, five regional pilots, with up to nine IPS teams, were nearing the conclusion of their project activities. Of the implementing projects covered in this study, two were exclusively integrated within the framework of psychiatric services, while the remaining three sites engaged in collaborative efforts with the public employment services (PES). In the latter case, two sites featured PES-funded employment specialists stationed at PES offices. One site was developed as an employment hub with shared workspaces with integrated employment services.

In Finland, healthcare legislation regarding psychiatric rehabilitation is relatively general, neither hindering nor facilitating the implementation of the IPS model. No national strategies, legislation, or funding mechanisms specifically focused on sustainment were in place. However, during data collection, the Finnish government launched a nationwide project to expand the IPS model, and the model was later included in the new government’s agenda, reflecting ongoing strategic commitment.

Notably, six months before the project’s conclusion and two years following the initiation of client work, a governmental change occurred: the responsibility for health and social care was transferred from municipalities and hospital districts to newly established regional authorities referred to as “Wellbeing Services Counties” [30]. The transition to the new governance structure was still in progress at the time of data collection for this study. However, the implementation projects were aligned geographically with these new administrative areas, and the national IPS project’s plan explicated an expectation that the Wellbeing Services Counties would take on the responsibility for financing the sustained use of the IPS model. The national project plan defined the pilots’ monitoring groups as responsible for making the arrangements for the sustained use of the model. The composition of the monitoring groups was defined by each project individually. A more detailed description of these groups can be found elsewhere ([31], p. 164).

### Sampling and data collection

Semi-structured included individuals, pairs, and groups were conducted by the first author, a male social science PhD University Researcher with extensive experience in conducting interviews. A purposive non-probability sampling targeted the implementing care units that were anticipated to provide the most information-rich and diverse perspectives [32]. The number of interviews was predetermined based on the expectation that data saturation would be achieved [33, 34]. We reached out to potential participants via email. While the target was set at 37 participants, eight invitees either declined to participate or were unreachable, resulting in a final analytic sample of 29 interviewees.

Based on the interviewees’ preferences, data were gathered in two primary settings: participants’ workplaces (*n* = 14) and online meetings (*n* = 15). Each interview session involved only the participant(s) and the researcher. The interview participants’ professional roles included outside experts (trainers, researchers) (*n* = 4), leaders from the implementing sites or PES collaborators (medical directors, nursing directors, administrators) (*n* = 11), project managers and team leaders (*n* = 6), and frontline workers (*n* = 8). The sample included both women and men, with careers spanning from mid to late stages. Demographic information was not collected to ensure privacy and confidentiality. The data were collected from May to August 2023.

The interview questions were developed based on the themes identified in a scoping literature review on the factors affecting the implementation and sustainment of the IPS model [6]. Consequently, pilot testing of the questions was considered unnecessary. The questions found in Additional File 1 covered mission alignment, organizational culture, work infrastructure, policies and laws, financing, training, evaluation, and future development plans. Interviews followed a semi-structured format, enabling flexible exploration of predetermined topics. Before conducting the interviews, the participants were provided with a written description of the research objectives, data handling procedures, information about the voluntary nature of their participation, and their rights as participants. The interviewer verbally reiterated his motivations and interests in the research topic and reminded the participants of the voluntary nature of their involvement before each interview. He interviewed each participant once. Interviews were audio-recorded, with a median duration of 72 min (from 48 to 120 min). Additional written notes were taken during the interviews. An external transcription service provider transcribed the audio recordings into standard Finnish. The transcribed materials were deemed factually correct and were not subjected to review or correction by the participants. Saturation was confirmed during the analysis, i.e., the sample captured the depth and diversity required to answer the research questions.

### Data analysis

We applied the six-step process of thematic analysis described by Braun & Clarke [35]; and chose an abductive thematic approach [36, 37]. The abductive approach combines deductive and inductive reasoning to align empirical findings with established theoretical frameworks and develop plausible explanations for observed phenomena. In this study, we began the analysis by familiarization with the data (step 1) and then generating initial codes (step 2) deductively, utilizing a list of categories identified in earlier research as important for IPS sustainment [6]. This deductive process of searching the themes (step 3) and reviewing the initial themes (step 4) led to the development of a three-pathway model that highlights the leaders’ relational position to the external context, organizational context and the implementation outcome (Fig. 1, above). Then, we inductively reviewed the results related to these pathways, defining the main themes and sub-themes based on their connection to the concept of leadership rather than the pathways themselves (step 5), and subsequently produced this report (step 6).

We categorized cases into two groups to improve reporting efficiency and reduce interviewees’ recognizability. Cases are classified as ‘with more robust sustainment strategies’ (n = 2) when the data indicate that leadership clearly communicated their commitment to sustainment and had secured at least the same number of employment specialist positions working under routine organizational conditions after the trial period. We refer to cases ‘with less robust sustainment strategies’ (n = 2) when these two conditions were unmet. One case demonstrated a ‘robust strategy’ in terms of commitment but did not fulfil the latter criterion. The results from their interviews could be reported in either category.

Selected quotes from the original transcripts were translated into English and presented in the results section to validate the analysis. We present coarsened data and use “she/her” pronouns for all participants to ensure anonymity, with alterations made judiciously to avoid distorting meaning.

The first author mainly conducted the analysis. The second author also familiarized herself with the data to ensure that the interpretations and the findings were derived from the data. All authors collaborated to review and define themes and produce the final research report. We adhere to SRQR and COREQ guidelines [38, 39] to maintain rigour and transparency in our research reporting. Atlas.ti program was used during the analysis process. ChatGPT (version 4) and Grammarly.com were used for proofreading purposes. Because of the manuscript’s lack of sensitive information, we did not return a manuscript draft to participants for comments or corrections.

## Results

We identified three qualitatively distinct leadership approaches in driving the sustainment process and navigating the dynamic interplay between internal and external contexts: Visionary, Proactive, and Collaborative Approach. The main themes, sub-themes, defining features, and their associations with the conceptual framework are presented in Table 1. The quotes supporting the themes, displayed separately for leadership with more robust and less robust sustainment strategies, are available in Additional File 2.

**Table 1:** Leadership approaches for IPS model sustainability: themes and findings

Main Theme (Leadership Approaches)	Sub-theme	Defining Features	Correspondence with the Conceptual Framework
**Visionary Approach**	Recovery Vision	Leaders prioritize sustainment goals by championing recovery-oriented principles, establishing strategic priorities, and fostering a shared vision to promote engagement and readiness for implementation.	Direct Leadership-Sustainment Pathway
	Goal Alignment	Leaders align IPS goals with organizational objectives while managing competing priorities, engaging stakeholders, and creating a supportive implementation climate.	Facilitator for Organizational Change
**Proactive Approach**	Forward-Thinking	Leaders initiate early sustainment planning by proactively addressing barriers and facilitators, ensuring resource readiness, and adapting plans for sustainment.	Direct Leadership-Sustainment Pathway
	Leveraging IPS Fidelity for Quality Improvement	Leaders use IPS fidelity reviews to drive organizational improvements by monitoring implementation outcomes, and enactingg quality improvements.	Facilitator for Organizational Change
**Collaborative Approach**	Monitoring Groups as Drivers of Sustainability	Leaders collaborate across organizational levels and with external stakeholders to drive sustainability by using monitoring groups to facilitate decision-making, enhance communication, and align organizational efforts for sustainment.	Facilitator for Organizational Change
	Boundary-Spanning Leadership	Leaders advocate for funding and align administrative policies with IPS sustainment goals by promoting resources, advancing governance policies to support IPS implementation, and addressing external conditions.	Moderator between External Contextual Factors and Sustainment

### Visionary approach

#### Recovery vision

Interviewees’ accounts highlighted differences in organizational approaches, with some leaders emphasizing recovery orientation while others focused more on traditional care practices with less emphasis on psychosocial outcomes using a team-based care approach. The organizations with the most robust sustainment strategies had already adopted the recovery approach as a strategic priority before the pilot. The IPS model could, therefore, be more easily placed into the existing psychosocial service repertoire in these organizations. In the first excerpt, a middle manager describes how the organization’s readiness, shaped by senior leaders’ alignment with the recovery approach, helped embed IPS into standard practices, and the second excerpt gives the employment specialist perspective:

*Promoting social participation is one of our agency’s goals*,* and the IPS model aligns directly with that objective.… Consequently*,* it has been one of our key performance indicators for the past two years. [Participant 1]*

*In our organization*,* the recovery approach is already integrated*,* so there has not been a need for separate enforcement of the model. [Participant 2]*

Leadership changes were noted as barriers to commitment to sustainment strategies. In the following excerpt, an employment specialist from an organization with a less robust sustainment strategy reflects on how a change in senior leadership led to moving away from the recovery-oriented approach. The comment shows how the leaders’ commitment to the project implementation phase could vary. Sustainment could be more challenging when becoming personalized to individual leaders and when direct links to organizational strategies were yet to be established.

*The decision to implement the IPS model was made during the tenure of the previous leader [in senior leadership]. She had been a strong driving force behind this*,* and there undoubtedly was a genuine intention to adopt the model.… The winds shifted*,* however. Initially*,* the approach seemed recovery-oriented*,* but later*,* other priorities began to take precedence. [Participant 3]*

Furthermore, sustainment was seen as dependent on a shared vision between senior and middle management. Several cases in this study, however, had one or more leadership tiers above the mental health care units, often including medical directors with backgrounds outside of mental health and limited familiarity with the recovery approach. Middle managers described negotiating competing priorities, such as balancing fidelity to the IPS model with corporate resource allocation. In the following excerpt, a middle manager discussed her position when buy-in and a shared vision had been achieved at the unit level but not at the corporate management level. These cases were in stark contrast to those care units with lower bureaucracy, having attained buy-in from the top leadership.

*It is like a puzzle. Without designated staff positions*,* it becomes crucial that my supervisor strongly supports the model. Her permission enables me to utilize the existing staff positions in this manner.… Should we fail to secure the designated funding*,* we will have to continue juggling these fragments of vacancies. I am prepared to attempt fixing them and somehow organizing the contracts. [Participant 1]*

The interviews did not reveal any direct objections to recovery approach principles. Instead, some leaders could be described as having an emerging commitment to implementing these principles with the IPS model. In such a scenario, while the sustainment strategy might not have been entirely in place, the interviews reflect the endpoint of the pilot as a moment where senior leaders were adopting the employment of psychiatric patients as a strategic goal for the organization.

#### Goal alignment

We found differences in whether leaders were perceived to use the IPS model as a broader quality improvement framework or to motivate staff to integrate IPS principles into everyday practices. The inclination to prioritize employment support as clinic-specific activities was at least emerging across most studied units and their teams. However, contrasts between the units with more or less robust sustainment could be found. In the first excerpt, an employment specialist from a site with more robust sustainment plans describes the clinical director’s assertion that the IPS model is an integral part of the psychiatric services, highlighting a leadership style that fosters the integration of employment specialists into treatment teams, a defining property of the model. The second excerpt, from a unit with less robust plans, discusses the need for a consistent message from the high leadership and the IPS model competing with other priorities, such as statutory services, which indicates a weaker or inconsistent climate for sustainment. An employment specialist highlights the asymmetry between organizational change and street-level practices.

*From the very beginning*,* our clinical director reminded me*,* “Remember*,* you are not some separate job coaching team; you are a part of the psychiatric services.” [Participant 4].*

*Most clinics and teams… are beginning to have employment support as the goal of their clinic-specific activities. However*,* it is not exactly consistent.… The shared message that comes from the high leadership is not at all clear.… [For them] there is much other stuff that takes up much effort*,* including that statutory services are not being implemented as they should be. [Participant 5]*

With senior leaders’ commitment to the IPS model as a vehicle for organizational change, project managers could show assertiveness. A project manager described herself as surprised by how well the new staff members were received in the psychiatric teams, although “*We did not give them many options. We approached them with our current proposal*,* expressing that this is the activity that we are now offering. Surprisingly*,* it worked quite well with the majority of the teams.” [Participant 6].* She had previously addressed the importance and function of leadership in this process. The leaders had committed to adapting the model and had communicated from early on that the idea was to implement a model that was about to be sustained. The assertiveness of middle leaders was perceived as necessary for integration during the project. In the following example, an employment specialist discusses proactive leadership and showcases its role in fostering a positive implementation climate. However, this example is drawn from a unit that had yet to secure staff positions at the end of the pilot. The example cautions against assuming that success in implementation during the pilot phase ensures sustained implementation. Instead, it emphasizes the connection between aligning the IPS model with the organization’s mission and culture and its sustained implementation.

*At our unit*,* the implementation has been straightforward. The nursing director had done a tremendous groundwork before the project even started; the nurses knew what the project was and what needed to be done. The nurses were already excited*,* and the whole team was excited about this project. [Participant 5]*

### Proactive approach

#### Forward-thinking

Leaders’ forward-thinking and personal commitment were identified in interviews with agencies that had more robust sustainment plans but these were absent in others. Their dedication to sustaining the service was particularly evident in their proactive efforts to make prior arrangements, effectively addressing challenges related to competing priorities. In a quote from a nursing director, the agency with a more robust sustainment strategy had imposed a strategy including sustainment plans from early on. She also highlights the importance of a shared agenda within senior leadership and integrating the model into the strategic framework of service provision.

*Of course*,* the fact that I had drafted the project application significantly impacted the implementation. The idea was developed jointly with our medical director. From early on*,* we recognized the reasons for needing such a service. Moreover*,* we had already contemplated how it would be sustained during the application phase if it proved successful. [Participant 7]*

In contrast, reactive leadership could prioritize other factors such as accountability in decision-making. Meeting accountability criteria was, however, perceived challenging as the healthcare information systems were to readily comply with the administrative information needs associated with the IPS model. Namely, the employment outcomes were not routinely recorded from the outset. Without standard monitoring mechanisms, feedback to leaders and the stakeholders in the monitoring group relied on informal communication and sporadic manual recording of the outcomes. Without a general strategic motivation for sustainment decisions, the lack of reliable monitoring data was considered problematic. The first excerpt demonstrates how an employment specialist perceived the pilot’s final stages under such conditions. The second excerpt presents an administrator’s perspective on the importance and function of monitoring within an administrative context.

*The project manager faced the challenge of reminding everyone in the monitoring group that this is a project and that projects have deadlines. The opportune time to contemplate the following steps is during the project to avoid any last-minute rush. Nevertheless*,* a rush did occur. The monitoring group was cautious about making decisions and preferred to wait until they had data. [Participant 5]*

*Our senior officer mentioned that she has yet to encounter any administrative leader for whom results are not essential when discussing funding*,* especially when considering sustainability. At that point*,* we appraise the results.… It has been quite confusing*,* to be honest. [Participant 8]*

#### Leadership leveraging IPS fidelity for quality improvement

The leaders’ eagerness to modify practices during the project period based on the recommendations received from fidelity reviews was one facet of the commitment to the IPS model sustainment. All units in this study had undergone two fidelity evaluations contracted by the national project. The first of the following quotes is from an employment specialist who expresses her positive perception of leadership making concrete changes in operations based on the received recommendations. The quote was provided shortly after a positive description of the medical director’s commitment and communication of the model to staff. In a contrasting example from a unit with less robust sustainment plans, a project manager discusses the ambivalence of conducting quality improvements in the face of project termination. The latter quote also highlights the two contrasting approaches to development activities: some organizations were developing their organizational practices using the IPS fidelity standards as a tool for that end, while in others, developing the IPS model in high fidelity was not perceived through the lens of organizational change.

*Over time*,* we have seen improvement. Initially*,* it was challenging because full participation in the care meetings was not possible.… Now*,* however*,* we can be fully present throughout the sessions. [Participant 9]*

*We made many changes according to the feedback. However*,* much more after the first round because the second evaluation round was quite recently*,* and there is no point in developing a terminating project. However*,* in the latest evaluation round*,* we received confirmation that we had been moving in the right direction. [Participant 10]*

Not only could the leaders’ perceptions of the IPS model as a tool for organizational development vary, but the organizational context itself was perceived important in guiding the overall implementation trajectory. Organizational or leadership changes were perceived to affect adaptability, leading to a lower implementation climate, including communication breakdown and low prioritization of the implementation effort. Employment specialists from a unit with a less robust sustainment strategy discussed how various co-occurring organizational contextual barriers jointly contributed to organizational non-adaptability.

*A new nursing director was appointed shortly after the pilot had started.… Also*,* there was an ongoing organizational change in the care unit during the pilot. So*,* there was no peaceful working environment where we could start thinking about the IPS model and how to build it better. As there was a constant struggle and people were not communicating with each other*,* we ended up being the last thing on the priority list. [Participant 3]*

At the end of the pilot period, PES withdrew from IPS development. PES did not view the IPS model as a quality improvement initiative at the organizational level; instead, they perceived it as diverging from their broader objectives. Interviewees highlighted challenges, including the need to adapt highly standardized employment services to address the diverse needs of the IPS model’s target group. This misalignment created tensions within the monitoring group, compounded by PES leaders’ engagement in major national employment service reforms. The project’s isolated nature, marked by communication breakdowns and low prioritization, further hindered collaboration.

In the first excerpt, an administrative manager described the disconnect as rooted in differing “ideologies” between the organizations. In the second excerpt, an employment specialist echoed this sentiment, highlighting the peripheral status of the IPS project within PES and its separation from mainstream operations.

*In the PES*,* the IPS pilot has practically been on the sidelines.… The pilot has been a separate entity within a small development team*,* and it has been oriented heavily towards the psychiatric care unit and adopting their ideology and ways of working.… To me*,* this IPS project was*,* frankly*,* just one among many other projects aimed at supporting and promoting employment.… This was just one of them*,* and I did not pay much attention to it [sustainment] then. [Participant 11]*

*None of the supervisors at the PES indicated they would have considered adopting the IPS model at any point in the process.… We were a completely separate entity. I did not even notice any intention of modelling this for standard customer service [Participant 3]*.

### Collaborative approach

#### Boundary-spanning leadership

The logic of the national IPS project’s program was to delegate the decisions regarding the sustainment of the IPS model to the regional and local decision-making levels. This approach was clearly articulated in the national project’s plan. Additionally, the national administration had not initiated any coercive policy mandates, legislative changes, or designated financial resources allocated by the administration to encourage the sustainment process also reflected this intent. The implementing units were expected to operate within the existing administrative framework.

The interviews revealed divergent views among leaders regarding who should be responsible for arranging the service. Senior leaders’ interviews revealed a high variability in perspectives on whether health services, social services, the national social insurance institution, or employment services should bear the responsibility for the sustained implementation of the initiative. In this context, interviewees highlighted the challenge posed by the absence of clear strategic guidance or a legal mandate. A specific challenge mentioned was the difficulty in positioning this initiative within the priority lists of mental health services, especially given that *“the statutory services in the wellbeing counties are not yet being realized as they should be”* [Participant 5] amidst ongoing national administrative reform. Consequently, leaders found themselves engaged in and facilitating two simultaneous organizational changes: one within their organizations, related to implementing the IPS model, and another external, involving broader contextual organizations. This challenge highlighted the absence of explicit role allocations and specific implementation strategies, as noted in the excerpt by an administrative manager:

*These goals*,* particularly social inclusion*,* employment*,* and rehabilitation*,* are high in our council’s strategy. So*,* yes*,* it fits in there nicely. Nevertheless*,* the actual question is whether the place is in specialized psychiatric care or some employment services*,* that needs to be clarified. [Participant 1]*

The national project set the stage for implementing the model with high fidelity, albeit only for the project’s duration. National experts organized seminars, inviting leaders to develop region-specific strategies for sustainment. Nevertheless, this approach had limitations. Not all pertinent issues could be tackled with the available stakeholder input, and interviewees reported challenges in integrating the IPS model with existing funding mechanisms. Without coercive elements in national legislation or strategies, designated funding, or established implementation frameworks, the locus of decision-making regarding sustainment shifted to local leadership. Two external experts emphasized this perspective:

*We already have the frameworks within the health care law that could support implementing the IPS model in psychiatric care throughout Finland. However*,* we still encounter challenges. There is a need for champions who inspire their communities to adopt the IPS approach. [Participant 12]*

*Indeed*,* it has emerged that there are institutional and structural factors at play. Some specific individuals are keenly interested in the IPS model and are committed to its promotion.… Their influence has been pivotal. As a result*,* it somewhat comes down to chance regarding who has been in those leadership roles. [Participant 13]*

The interviews revealed two contrasting approaches to participating in external organizational transitions: active and passive. In one of the pilots, the leaders seized the opportunity to integrate the IPS model into the regional governance strategy amidst the organizational change. They were actively involved in preparatory committees, reinforcing their efforts to sustain the number of employment specialists for designated staff positions. The nursing director involved in the process observed that the approach mirrored the processes in the old organizational structure and did not involve building consensus with administrators or politicians who would champion the model. Discussing the strategic integration of the IPS model, she noted:

*If a regional council or a government politician finds a connection to this and starts lobbying for it*,* it can have a tremendous impact*,* particularly in securing additional funding. Such cases do occur occasionally.… However*,* the IPS model did not go through that way. I am not saying it was opposed*,* but they probably were unaware of the details. [Participant 4]*

The absence of strategic collaborative arrangements between the funders and the implementing agencies could challenge the decision-making regarding sustainment. Several interviewees discussed their difficulty establishing solid connections with the expected funding authorities and setting clear terms or contractual agreements (along with specifying auditing criteria). A project manager shared her perception of the challenges the monitoring group faced in creating a structure for building sustainability, as mandated by the national project:

*The fundamental issue is not whether it is evidence-based or not*,* but instead… there is a lack of incentives for stakeholders. It manifests when a client gains employment: one entity funds the initiative while the financial benefit is distributed among many parties. Consequently*,* there is too little economic interest for anyone to invest in it. [Participant 10]*

To conclude, without administrative strategies and their implementation, the responsibility for sustaining the service was devolved to the level of the local agency leadership.

#### Monitoring groups as drivers of sustainability

Monitoring groups were established in all projects during the pilot period, with sustainment planning mandated as one of their core functions. Despite this, neither agencies with more robust nor those with less robust sustainment plans reported that these groups were utilized effectively for this purpose. This gap persisted even though the national program had facilitated efforts to support sustainment planning. Instead, these groups reportedly lacked decision-making authority. Furthermore, the interviewees perceived these groups as lacking a clear focus on sustainability, functioning more as information-sharing platforms than proactive decision-making bodies. A nursing director from an agency with a more robust sustainment strategy did not perceive this necessary as a limitation:

*After all*,* the only thing needed for sustainment is having a cost center with a budget*,*… for which our authorities are certainly sufficient.… The most difficult thing here was daring to decide to cut back some of our existing activities so that we could establish this model. [Participant 7]*

However, the interviews revealed that monitoring groups were not widely utilized as a steering mechanism for ensuring sustainability. In contrast, groups were described to have limited impact due to disengagement of senior leaders and lack of mission clarity. A member from one agency with a less robust sustainment plan observed:

*At first*,* [the top manager] was the chairperson*,* but then not much later [a lower-tier manager] became the chairperson*,* and soon [the top manager] was not in those meetings anymore.… It has not been easy for the project manager*,* as steering-group members did not really take any stand. They were such high-level people that they might not have wanted to get too involved in this project. While they acknowledged the potential benefits of establishing the IPS model*,* believing it to be a valuable addition*,* their engagement did not extend beyond this initial endorsement. [Participant 8]*

The interviews revealed two qualitatively distinct functions of monitoring groups as steering mechanisms: first, serving as a platform for providing feedback to external stakeholders and ensuring sustainability, and second, acting as a platform for developing and refining implementation strategies within the organizational boundaries. A project manager described transitioning the monitoring group from a broad stakeholder forum to smaller, task-oriented teams focused on agency-level implementation and sustainment planning. Yet, the change left unresolved the issue of integrating the service as a prioritized component within the broader service structure.

*The positive development is that a new steering group has been established*,* which is responsible for supporting further integration and development. This steering group includes all line managers and nursing directors from divisions with outpatient clinics. [Participant 10]*

## Discussion

### Summary and reflection of findings

The study described integrating the Individual Placement and Support (IPS) model within psychiatric rehabilitation services in Finland, focusing on the leaders’ role in driving the sustainment process and navigating the dynamic interplay between internal and external contexts. We identified three Leadership Approaches for IPS Model Sustainability that differentiated agencies with more robust sustainment strategies from those with less robust ones. The Visionary Approach encompassed strategies and actions related to championing recovery-oriented principles, fostering organizational readiness, and aligning IPS with overarching organizational goals to ensure integration into long-term practices. The Proactive Approach was characterized by early and deliberate planning to address potential challenges and ensure a smooth transition from the project phases to sustained operation. Proactive leaders leveraged IPS fidelity evaluations as a tool for continuous quality improvement, using the findings to refine practices and maintain adherence to IPS standards. The Collaborative Approach included facilitating the sustainment of the IPS model within governance structures by actively embedding it into the strategies of administrative bodies.

Our results align with the IPS model’s originators’ early observations, emphasizing the necessity of solid leadership commitment throughout the organizational levels to implement the IPS model’s dualistic objectives successfully. Such coordinated efforts may be required because leadership for IPS model sustainment involves facilitating service integration as an organizational principle and prioritizing employment support as a core service in mental health care [39]. The IPS model could be perceived as a method for implementing this broader strategy, which was more challenging in more complex organizational structures, as noted in earlier studies [10]. In our data, agencies with the most robust sustainment strategies were characterized by senior leaders who were committed to recovery principles, this buy-in potentially representing a complementary finding to the prior evidence linking leaders’ engagement and commitment to quality improvement with sustainability [13]. The interviews suggested that leadership is important in establishing common goals both within and among participating organizations. Not perceiving the IPS model as a mechanism for implementing broader strategies was most evident in the collaboration between public employment services and healthcare, a collaboration often reported as problematic [15, 21]. In the project covered in this study, employment services decided to withdraw from any future operations following the project period. While the recovery approach may facilitate organizational change within psychiatric care settings, collaborations involving organizations with diverse missions and distinct legal mandates likely require similar goal alignment, as the leaders could perceive the IPS model as conflicting with traditional medical or vocational services [40, 41].

Our results suggest that external contextual factors—such as funding, policies, laws, and support functions—pose contextual challenges for leadership particularly in relation to the model’s sustainment processes. While a national, government-sponsored project was generally perceived as providing sufficient resources for developing and implementing a high-quality service, and a relatively lax legislative framework did not facilitate nor hinder the project, these items emerged as highly crucial for the service’s sustained use. The model originators have long stressed administrative collaboration to address such challenges [19]. Noel et al. [12] suggest that the IPS model sustainment is likely a synergistic function of leadership at levels of learning collaboratives, state governments, and local programs. Governments have begun to counteract these challenges with strategic initiatives [6]. Such structured collaboration was absent in this study, despite the establishment of monitoring groups that included participants from agencies considered responsible for sustainment. The role of these groups in addressing sustainability was limited in all cases, and this challenge was further compounded by ongoing administrative reforms. Many interviewees highlighted the uncertainty about which administrative branch should ultimately assume responsibility for oversight, and discussions about long-term funding were not initiated early on—a factor identified as important for sustainability in previous studies [15]. Moreover, none of the groups succeeded in establishing effective feedback mechanisms to funding authorities, a recognized facilitator of IPS model sustainability [13]. These challenges lead the service’s sustainment to largely depend on local leaders’ decisions. Our results suggest that this heavy reliance on championing local leadership could introduce variability to sustainment strategies due to variations in individual commitment. This study supports the idea that structured strategies at the national or regional administrative levels can aid leaders in transitioning to sustained services by facilitating the integration of the service model into the broader service structure and helping navigate leadership changes.

As a distinct research focus, sustainability has received less attention in both the IPS model [6] and EBPs in general health service delivery [7] research, and our findings highlight focal points for future research. First, confirming these findings in different settings and through diverse methodologies. Second, identifying barriers and facilitators unique to sustainability, such as leaders’ early commitment to sustainment planning and organizational safeguards to mitigate the potential negative impacts of leadership transitions. Third, further research is needed to explore the interactions between leaders and stakeholders across different administrative levels. Lastly, the role of national and regional policies in supporting leadership for sustaining IPS implementation warrants investigation. A broader range of policies, even those not directly tied to the IPS model, may shape organizational contexts in ways that influence leaders’ capacity to sustain services effectively.

### Limitations and strengths

This study has several limitations. The qualitative nature of this research precludes any claims of causality between the identified factors. The relatively small number of cases (*n* = 5) and the specific context of the Finnish healthcare system may limit the transferability of the findings. Particularly, the results must be interpreted with care, as the study’s analytic terms, “more” or “less robust sustainment strategies,” may not be linked to measurable evaluation criteria such as penetration or reach, limiting the credibility of the findings. The study’s focus and data collection were restricted to the agency-level leadership, excluding detailing administrative decision-making processes and a nuanced analysis of monitoring groups, which limit transferability of the findings. Using a conceptual framework for organizing qualitative data bears a risk of over-interpreting interviewees’ perspectives to fit the researcher’s preconceptions. To counter this risk and ensure the accuracy of our findings, we sought and reached concurrence on the data representation between two authors. Not conducting member checking may hinder credibility and confirmability of the findings. To reinforce the dependability of our results, we have made an effort to be transparent about the research process and have adhered to standardized SRQR and COREQ reporting guidelines.

The study highlights the importance of leadership as a driving force in sustaining EBPs. This study addressed knowledge gaps in EBP implementation research by emphasizing sustainment as a core implementation outcome, positioning leadership as its central driver, and highlighting contextual factors as major influences for sustainability. The conceptual model developed in this study demonstrated plausibility.

## Conclusion

This study contributes to our understanding of the leadership’s role in sustaining the Individual Placement and Support model at the conclusion of a multi-site development project in Finland. We identified three Leadership Approaches for IPS Model Sustainability that distinguished agencies with more robust sustainment strategies from those with less robust ones. Additionally, we introduced and empirically explored a conceptual model that may inform future research and practice.

## Supplementary Information

Supplementary Material 1.

Supplementary Material 2.

## Data Availability

The raw data are protected and are not available due to data privacy laws.

## Abbreviations

EBP: Evidence-Based Practice
IPS: Individual Placement and Support
PES: Public Employment Service
