# Supplementary material for: Leadership in sustainment of Individual Placement and Support model: a comparative case study in Finland
Source: BMC Health Serv Res. 2025 Mar 4;25:334. doi: 10.1186/s12913-025-12495-1 (PMC11877839; doi:10.1186/s12913-025-12495-1)
Supplement: Supplementary file 1 — Supplementary Material 1. [file 12913_2025_12495_MOESM1_ESM.pdf]

Additional File 1

INDIVIDUAL PLACEMENT AND SUPPORT AND INSTITUTIONAL STRUCTURES -PROJECT

INTERVIEW QUESTIONS

I BACKGROUND QUESTIONS

• Describe your job duties, especially duties related to the IPS model.

• How do you feel about the IPS development project, and how would you describe your experiences with it?

• Have you participated in other development projects? Have they been evidence-based, and how does the development of the IPS model compare to them?

• What factors led to participation in your organisation's IPS project, and why did you join?

• Looking ahead, what are your plans with IPS? Are you considering continuing with it in the future? If so, could you please describe your plans and any decisions made?

II. SERVICE MODEL

• How well do you know the scientific basis of the IPS model? (customer results, quality criteria, successful implementation)

o What does recovery orientation mean to you?

• Where did you get information about the IPS model, and was the information you received sufficient?

• How clear do you think the IPS model is?

• How easy has implementing the IPS model been, and which factors have influenced your views?

• How well does the service, according to the IPS model, combine with the whole formed by other services?

• Do you think the IPS service is available to all patients of the treatment units that participated in the project? How is the matter monitored?

III. IMPLEMENTATION

Organisational goal setting

• How has the IPS model responded to customers' real needs and situations?

• Has the service provider's understanding of the operational goals changed after introducing the IPS model?

• How has the development project affected the matter?

• Has the model conflicted with the goals of existing practices?

Professional attitudes and norms

• Has the IPS model been perceived as legitimate in organisations?

• What factors have influenced these experiences?

• What kind of atmosphere has prevailed among the various actors during the IPS project?

• Are you aware that the goals of IPS have influenced the professional attitudes and norms of healthcare professionals and other professional groups?

Organisation of work

• Is customer work in all units teamwork?

• Are the employment specialists integrated into the teams?

• Which factors are the biggest obstacles to integration? What factors related to work organisation have you come across?

• How well has the cooperation between different professional groups and actors gone during introducing the IPS model?

• Have you encountered cooperation-related problems, and how have you tried to solve them?

• What is your experience with human resources to implement work according to the IPS model?

• Has staffing been sufficient?

• How do you evaluate the competence of the staff? Is there room for improvement?

• Has there been staff turnover? How has it affected the implementation?

III. SYSTEM LEVEL (Policy and legislation)

• What kind of national mental health strategies, guidelines and agreements exist to support the implementation of IPS?

How well do IPS principles fit with other national policy objectives, such as social inclusion and participation?

• IPS has been part of treatment guidelines since the beginning of the 2010s, so why is it introduced only now?

• Do you see that the development work of the IPS model promotes the dissemination of practices and broader system reform at the national and regional levels? Which factors are in favour and which are against?

• How do you assess the suitability of the IPS model to the legislative framework regulating the operation of service providers? Which factors promote or hinder the adoption of the model?

• Is the IPS model compatible with the service structure of national mental health services?

• What kinds of development needs do you identify in the system?

• How do you feel about the incentives to implement the IPS model, such as financial support, training, and fidelity evaluations? Are they sufficient, and where are the possible problems?

A) During the development project

B) As a sustained practice

• How is the implementation planned to be organised after the project period? Who are the responsible actors, and what strategies are needed?

IV. STRATEGIES AND MANAGEMENT:

• How well do you know the mental health strategy of which the IPS projects are a part?

• How satisfied are you with the activities of the monitoring group gathered around the project?

• How could the monitoring group's performance be developed?

• Do you think the strategy and its implementation have been implemented consistently?

• Could you describe the implementation strategy of the development project?

• Do you think the strategy and its implementation have been consistent?

• Do local service providers / you have strategies for sustained operations?

• How has the development project supported the formation of local sustainment strategies?

• How satisfied are you with the activities of the monitoring group gathered around the project?

• How could the monitoring group's performance be developed?

• Has securing sustained funding and creating a sustainable funding model been part of the development work? (National, regional, and local level)

V. TRAINING AND EVALUATION

• Have you participated in training related to the development of IPS? In what role and how often?

• How have you experienced the training related to IPS development? Do you think their content has been sufficient?

• How systematic methods have been? What are the experiences of training?

• How will employee training be carried out when the operation is sustained, and the personnel changes are likely?

• How do you see the role of scientific research as part of development work? Has the work been successful as planned? What would you do differently?

• How is the operation of IPS monitored within THL/nationally? What metrics have been used?

• How would you evaluate the implementation of the fidelity evaluations and the recommendations received?

• Did the fidelity evaluations affect your operations? How?

• Did the development needs found in fidelity evaluations correspond to your understanding of operational development needs?

• According to your knowledge, does THL or another operator intend to conduct evaluations after the project period?

IV. SUSTAINMENT

• What kind of steering structure supporting the IPS model have you planned or introduced?

• What is the role of research and development?

• Do you have a financial strategy for the future?

• Who has been responsible for taking these decisions forward?

• What agreements exist between service providers and funders regarding performance reporting?

V DEVELOPMENT NEEDS

• If you could decide about the IPS model, would you change something in the organisation or implementation of the services?

• If we consider the implementation of the IPS model in Finland / your organization in three years. The IPS service is implemented optimally.

o What things have changed compared to today?

o Who or what factors have helped you reach your goals?

o What kind of support have you received, and from whom?

• How do you see the role of IPS in the future service structure of your Wellbeing County?

• We have now reached the end of the interview. Can you still tell me if you missed something important during the interview?
