# Supplementary material for: Leadership in sustainment of Individual Placement and Support model: a comparative case study in Finland
Source: BMC Health Serv Res. 2025 Mar 4;25:334. doi: 10.1186/s12913-025-12495-1 (PMC11877839; doi:10.1186/s12913-025-12495-1)
Supplement: Supplementary file 2 — Supplementary Material 2. [file 12913_2025_12495_MOESM2_ESM.pdf]

Additional File 2: Interview Quotes Supporting the Thematic Structure, with Crosstabulation by Compared Cases

**Themes**	**Leadership in agencies with more robust sustainment strategies**	**Leadership in agencies with less robust sustainment strategies**
**Main theme: Visionary Approach**		
Recovery Vision	If the care agency already embraces a strong recovery-oriented mindset as a core of its ethos, it significantly contributes to the process. Arguably, this is among the most crucial factors: the leaders being committed to the values that support the right of individuals to participate fully in the workforce. Furthermore, it involves the belief in their capacity to work. Such values and attitudes could be seen central, paving the way for the readiness to adopt and implement this model. [Outside expert 1]	The decision to implement the IPS model was made during the tenure of the previous leader [in senior leadership]. She had been a strong driving force behind this, and there undoubtedly was a genuine intention to adopt the model. ... The winds shifted, however. Initially, the approach seemed recovery-oriented, but later, other priorities began to take precedence. [Employment specialist 1]
	Support from management varies in intensity. While every initiative likely received some management endorsement to get off the ground, robust, unwavering support is the thing that truly makes the difference. [Outside expert 2]	It's clear that we need to elevate its [recovery orientation] importance across the board. Emphasizing recovery orientation is not just a goal; it's a significant part of the cultural shift we strive to achieve and embed more deeply within our practices. This shift may represent a substantial cultural transformation: moving from a purely nurturing approach to prioritising and championing a recovery-oriented perspective. [Medical director 1]
	Some leaders place a high value on new models and actively seek to introduce them to the organization. In that case, the workplace culture has likely already been assumed the appropriate values and atmosphere. In other words, the community is more receptive to such operating models. When we discuss recovery orientation, we	Q: How would you rate the support from an organizational perspective? A: The leadership hasn't communicated much directly; everything comes through in stages. Our nursing director has been absolutely 100% supportive of us, and her support has been evident to me. However, we haven't received such support from the higher management. [Employment specialist 1]

	essentially talk about adopting specific tools and practices based on these principles. [Outside expert 2]	
	In our organization, the recovery approach is already integrated, so there has not been a need for separate enforcement of the IPS model. [Employment specialist 2]	The employment specialists arriving at Site D have depended on how each individual employee interprets and experiences their presence. What has been lacking is a clear vision from the top directors, indicating that their role is integral to the recovery-oriented approach. [Project manager 1]
	It is like a puzzle. Without designated staff positions, it becomes crucial that my supervisor strongly supports the model. Her permission enables me to utilize the existing staff positions in this manner. … Should we fail to secure the designated funding, we will have to continue juggling these fragments of vacancies. [Nursing director 1]	There might be what I would describe as a cautious commitment to the model. It is currently seen as important and meaningful. However, we have not secured additional resources for it, which also reflects the situation. [Nursing director 2]
	Recovery orientation is foundational; it's the starting point for everything we do. The recovery orientation should underpin your work if you work within our psychiatry. I have always been a strong advocate for this approach. [Project manager 2]	Nursing director 2: I have discussed the issue with my supervisor, the leading nursing director. There have also been discussions among our senior nursing colleagues. However, the strategic goals should come from someone higher than us. I wish there were clearer guidelines. Nursing director 3: We have certain resources at our disposal. We have a certain obligation to utilize them in the best possible way for the patient. However, the larger decisions, or those concerning additional resources, are made at a higher level
	Navigating the complexities of healthcare priorities is a reality that demands prolonged, diligent effort and a clear vision and focus. [Medical director 2]	

	Q: Are there opinion leaders at the management and expert level of the organization? Did I understand correctly that [Medical director A] is very committed.<br>Employment Specialist 3 and Employment Specialist 4: Yes.<br>Q: Can you think of any others?<br>Employment Specialist 3: Perhaps it's unique to [Medical director A], from our perspective. She is the one who pushes things forward.	
	The positive impact of our management's support is evident. When a new initiative is introduced from the top, it's received much more favourably than without such backing. This highlights the significant role that management support plays in the acceptance and success of new ideas within the organization. [Project manager 2]	
	The best place for everyone is in the community. It means removing hindrances and helping the patients to typical work arrangements and conventional communities where everyone else is. [Medical director 2]	
Goal Alignment	Some participants have embraced the model with the understanding that its adoption is a foregone conclusion, viewing the project as simply a necessary procedure to undergo. Conversely, others perceive it as a project among others, undertaken because funding was available, and adopt a 'wait and see' approach regarding its outcome in two or three years. [Outside expert 2]	In the projects, particularly in Location C, there was a significant turnover among the medical staff. This turnover necessitated starting the process of establishing and justifying the project from scratch. It poses a considerable risk when the initiative is too closely associated with one individual or a few people within the medical community. The responsibility for the project should ideally be distributed more broadly within the organization or anchored at a higher level to mitigate this risk. [Outside expert 1]

	Sites A and B are undoubtedly the standout performers. At Site A, the success can largely be attributed to the to a certain director, who has played a crucial role. Similarly, in Site B, the medical director has been instrumental. They recongize this model should have been implemented 20 years ago. [Outside expert 3]	Most clinics and teams … are beginning to have employment support as the goal of their clinic-specific activities. However, it is not exactly consistent. … The shared message that comes from the high leadership is not at all clear. … [For them] there is much other stuff that takes up much effort, including that statutory services are not being implemented as they should be. [Employment specialist 1]
	It fundamentally comes down to a decision by the management. Adopting the model was a deliberate choice we made. [Nursing director 5]	Q: Is the employment of your clients part of this organization's goals? A: Not yet. We may be moving in that direction, but it depends on whom you ask, so the responses will likely vary significantly. [Employment specialist 1]
	From the very beginning, our clinical director reminded me, "Remember, you are not some separate job coaching team; you are a part of the psychiatric services." [Employment specialist 2].	A: Indeed, there has been questioning from the management's side regarding whether this truly falls within the scope of psychiatry or if it should be someone else's responsibility.<br>Q: Who from the management has communicated in this manner? A: Members of the steering group [Employment specialist 1]
	We did not give them many options for the care teams. We approached them with our current proposal, expressing that this is the activity that we are now offering. Surprisingly, it worked quite well with the majority of the teams." [Project manager 2].	If we had necessary funding, I would ensure proper staffing, establish a team, and so on. However, such an initiative isn't worthwhile under current circumstances. [Medical director 3]
	While it may not be formally documented, the emphasis on recovery orientation and the focus on integrating into working life, education, and society is already evident at	The branch manager has also spoken favourably about the model. However, perhaps without taking a clear stance indicating that this is the vision and direction we are heading towards. [Project manager 1]

	the upper management level. It is indicated by their goal-oriented approach to these matters. [Project manager 2]	
	Obtaining this requires long, persistent work, along with a clear vision and focus. One must concentrate on certain things and persistently push them forward. It demands prioritization because there are many other things besides this particular model in the treatment guidelines. [Medical director 2]	I began discussing the necessity for proper planning regarding the sustainment process, emphasizing that we must refrain from assuming this will be sustained on its own; it must be connected to organizational goals. I have attempted to convey the message to the steering group, highlighting that this initiative is not clearly linked to anyone's objectives. ... The goals of our organization do not explicitly encompass work and employment support. [Project manager 1]
	We have a long tradition in psychiatry of multi-professional working groups and collaborative practices, so our approach to treatment is not solely focused on care. Nurses and doctors are accustomed to the fact that rehabilitation is a significant component of treating patients with psychosis. It is recognized that both treatment and rehabilitation are integral to this process, and there is a keen interest in these aspects. [Nursing director 3]	The key considerations include how top management values the initiative, how it is articulated and documented, and how resources are allocated, along with granting permission to proceed and ensuring effective communication. [Nursing director 4]
	After all, the only thing needed for sustainment is having a cost center with a budget, ... for which our authorities are certainly sufficient. … The most difficult thing here was daring to decide to cut back some of our existing activities so that we could establish this model. [Nursing director 4]	At no stage has any person responsible for the PES explicitly stated that the sustainability of the IPS model was a consideration. I fully understand that, given the statutory responsibilities the office already holds, it's not clear to me why they would suddenly divert funds to implement this. I perceive it was seen as an additional burden. [Employment specialist 1]
**Main theme: Proactive Approach**		

Forward thinking	At Site A, the adoption of the new service was swift, signifying an immediate embrace of change. From an early stage, the IPS model found its place within their organizational structure and arrangements. Its integration was promptly established. [Outside expert 2]	Site B's decision regarding the project's future was made really late, creating an awkward situation. Many of us believed that the initiative would become sustained and that they could continue as IPS employment specialists. [Employment specialist 1]
	Sites A and B have demonstrated a clear commitment and long-term dedication from the initial planning stages to the piloting implementation and subsequent continuation efforts to ensure successful integration. Consistently, the same group of individuals influences these processes behind the scenes. [Outside expert 3]	The time-span of planning has been short. The project manager faced the challenge of reminding everyone in the steering group that this is a project and that projects have deadlines. The opportune time to contemplate the following steps is during the project to avoid any last-minute rush. Nevertheless, a rush did occur. The monitoring group was cautious about making decisions and preferred to wait until they had data. [Employment specialist 1]
	Of course, the fact that I had drafted the project application significantly impacted the implementation. The idea was developed jointly with our medical director. From early on, we recognized the reasons for needing such a service. Moreover, we had already contemplated how it would be sustained during the application phase if it proved successful. [Nursing director 3]	Our senior officer mentioned that she has yet to encounter any administrative leader for whom results are not essential when discussing funding, especially when considering sustainability. At that point, we appraise the results. ... It has been quite confusing, to be honest. [PES director 1]
	One of the strengths is likely the fact that it was recognized within the service line. When it became apparent that such a project was available, it was deemed worthwhile to pursue because the management level had already contemplated how the model fit into the overall service palette and viewed it as a component of the rehabilitation process. [Project manager 3]	When we began, a project plan was in place, and we adhered to it. My main concern, however, was its omission of any post-project considerations. What were the objectives following the project's conclusion? Ideally, this should have been outlined from the start, providing everyone with a clear understanding of how the initiative would proceed. Unfortunately, this decision was deferred until the last

		moment, by which time the original champions were no longer involved. [Project manager 4]
		The desire and will to truly examine what we could do internally in a new or different way was lacking previously. It may have only begun to emerge only at the end of the project. [Project manager 4]
		More awareness has begun to emerge in this final phase of the project. Perhaps this issue could have been considered earlier during the project's progression. That way, we wouldn't be facing this question of resources just as we're nearing the project's conclusion. [Nursing director 4]
		If we decide to adopt this admittedly good model, the implication is that we might have to abandon some old ways of working, and it would involve prioritization. However, I still don't have a direct answer regarding whether we will prioritize the IPS model. [Medical director 3]
Leveraging IPS Fidelity for Quality Improvement	In these experiments, a noticeable difference emerges based on their inception - some were initiated purely as projects or trials. In contrast, others were aimed at establishing permanent activities right from the start. This fundamental distinction often bypasses the notion of a transitional phase, setting a clear objective to integrate the initiative permanently. These differences are evident and have also influenced the perception of challenges encountered. [Outside expert 3]	The announcement was made on a very tight schedule, indicating the arrival of this new initiative. There was a lack of staff involvement, leaving no opportunity to deliberate how we might like to structure this collaboration. So, this was impulsively imposed from above without much consideration for ground-level input. [Employment specialist 1]

	Management support is crucial. Without it, progress is impossible. This principle applies to IPS model and all practices being adopted. Our project exemplified this well; it was particularly evident in [Location A], where the initiative to apply for the project came directly from the management, which also took the lead in writing the application. Clearly, with management's support as a cornerstone, every aspect of the project becomes significantly smoother. [Outside expert 2]	The project faced significant challenges, primarily because it was timelimited and involved two large, inflexible organizations. This situation led to the primary challenge: realising that the current approach wasn't viable. [Employment specialist 1]
	Over time, we have seen improvement. Initially, it was challenging because full participation in the care meetings was not possible. … Now, however, we can be fully present throughout the sessions. [Empoyment specialist 3]	The arrangement has been such that the team leader oversees the employment specialists, yet both the team leader and the specialists report to a separate supervisor from our administration. This places me in a unique position as a development manager. ... We have had quite a confusing management structure on our end, too. [PES manager 2]
	The IPS operating model aligns well with the requirement to demonstrate a clear service pathway tailored to the patient's needs, from acute care to rehabilitation. We should be able to substantiate this pathway to anyone who inquires. [Nursing manager 4]	We made many changes according to the feedback. However, much more after the first round because the second evaluation round was quite recently, and there is no point in developing a terminating project.<br>However, in the latest evaluation round, we received confirmation that we had been moving in the right direction. [Project manager 3]
		A new Nursing director was appointed shortly after the pilot had started. ... Also, there was an ongoing organizational change in the care unit during the pilot. So, there was no peaceful working environment where we could start thinking about the IPS model and how to build it better. As there was a constant struggle and people were not

		communicating with each other, we ended up being the last thing on the priority list. [Employment specialist 1]
		In the PES, the IPS pilot has practically been on the sidelines. … The pilot has been a separate entity within a small development team, and it has been oriented heavily towards the psychiatric care unit and adopting their ideology and ways of working. … To me, this IPS project was, frankly, just one among many other projects aimed at supporting and promoting employment. … This was just one of them, and I did not pay much attention to it [sustainment] then. [PES manager 3]
		None of the supervisors at the PES indicated they would have considered adopting the IPS model at any point in the process. … We were a completely separate entity. I did not even notice any intention of modelling this for standard customer service [Employment specialist 1]
		Manager A, who also wrote this application on our behalf at the time, is a psychosocial services developer. She strongly advocates for the IPS model. However, she isn't in charge, and her involvement has been somewhat informal. [Project manager 4]
		Q: What are your experiences and views on these fidelity evaluations? Has the information gained from them been sufficient and helpful? A:From what I've been able to observe, yes, I've encountered some of the same challenges as other professional groups. The limitation of my own working time and how much of it I've been able to dedicate

		to this follow-up work has been a personal challenge for me. [Medical director 3]
		I soon realized that we were perceived as a separate project, meaning we were treated as one stakeholder group. As a result, I also had to adjust my own perspective, acknowledging that this isn't an organizational development project. Instead, we were seen as an external actor trying to integrate into the polyclinics.[Project manager 1]
		The second round of evaluation took place half a year before the project ended. At that point, developing a project nearing its conclusion made little sense. However, during this evaluation round, we received confirmation that we had moved in the right direction. [Project manager 1]
		The IPS project has been treated as a separate initiative within our office. I understand that it has not fully integrated or found a foothold within the office during this project period. It has operated in isolation, operating from the small development team. [PES manager 3]
**Main theme: Collaborative Approach**		
Boundary-Spanning Leadership	Now that we are in the wellbeing couties, budget cuts seem to prompt immediate reconsideration of the feasibility and necessity of our services as the funds are tight. We navigated through our health and medical care management groups and succeeded in standardizing this new initiative [in regional council's strategy]. As a result, it has been established as a sustained activity. [Medical director 4]	The transition to welfare areas has significantly complicated matters. This substantial structural change directly impacts funding, affecting the sustainability of IPS. While management's support, desire, and internal decision-making power are critical, they cannot compensate for the absence of funding. In essence, without funding, these efforts face significant challenges. [Medical director 3]

	The wellbeing county reform presented a favorable opportunity since the organizational structure was in flux. By that time, we were already committed to adopting the IPS model. I was selected for a service development working group. During this phase, we successfully incorporated the IPS model into our preparatory materials ahead of the election of new politicians. When the politicians finally ratified the decision, the model's sustainability was assured. [Nursing director 3]	The message from management is far from clear, but I believe this is due to our current challenges. Implementing statutory services is not up to par, among other pressing issues that demand significant attention and resources. [Nursing director 2]
	If a regional council or a government politician finds a connection to this and starts lobbying for it, it can have a tremendous impact, particularly in securing additional funding. Such cases do occur occasionally. … However, the IPS model did not go through that way. I am not saying it was opposed, but they probably were unaware of the details. [Nursing director 3]	However, there remains some confusion regarding who ultimately holds responsibility for implementing and sustaining. Which organization should take ownership? This ambiguity is partly due to legislative complexities and, on the other hand, to the evolving nature of wellbeingties. ... In my view, it's still unclear to everyone involved whose domain this kind of vocational rehabilitation falls into, particularly when it overlaps with healthcare. Consequently, responsibilities remain fragmented and it's still being determined which sector should oversee these efforts. [Medical director 1]
		Unfortunately, PES legislation and the constraints of unemployment insurance significantly direct actions in a certain way, so implementing the best practices from various experiments in the long term is challenging. [PES director 3]
		The fact that the service process for PES clients is so strictly defined and regulated at the legislative level, combined with the insufficient resources allocated to the PES office in accordance with that process, only allows for a very

		formulaic service, which is far from being innovative. [PES director 3]
		Unfortunately, the IPS project coincided with a period of significant organizational reform led at the national level, which resulted in complete disruption within our organization. [PES director 3]
		At no point has any of my supervisors at the PES stated that introducing the IPS model as sustained service was being considered. I understand their viewpoint. When the office already has statutory responsibilities, why would they suddenly allocate funds for what seems like an additional task?<br>[Employment specialist 1
Monitoring Groups as Drivers of Sustainability	Q: How satisfied have you been with the IPS steering group's activities? A: If asked to rate on a scale from one to ten, I would give it at least an eight and a half, if not a nine. The minor drawbacks include occasions when people have more pressing meetings. However, we have been able to request recommendations and feedback. It has been a tool for this project, which has promoted our activities through its networks of cooperation and influence. [Project manager 3]	At first, [the top manager] was the chairperson, but then not much later [a lower-tier manager] became the chairperson, and soon [the top manager] was not in those meetings anymore. ... It has not been easy for the project manager, as steering-group members did not really take any stand. They were such high-level people that they might not have wanted to get too involved in this project. While they acknowledged the potential benefits of establishing the IPS model, believing it to be a valuable addition, their engagement did not extend beyond this initial endorsement.<br>[PES manager 2]
	Having participated in the IPS steering group at Location A, I've noticed that the discussion culture is quite distinct. The chairperson fosters a very interactive environment, encouraging the group to discuss and reflect. This contrasts	Perhaps what was needed in these situations as well was the acknowledgment of not knowing, such as being unaware of another organization's activities, advantages, and the capacity to offer support. This openness could have

	sharply with our steering group, where deliberation was virtually nonexistent. [PES manager 1]	facilitated better collaboration and understanding. [PES manager 2]
		They've been sharing updates on progress, but they haven't been effective in advancing the issue themselves. Discussions on effectively implementing or moving forward with the initiative have taken place in entirely different forums, not within these steering groups. [Medical director 1]
		If I could approach it differently, I would recognize that fostering the engagement of people in leadership positions or steering group representatives is also requires robust facilitation. [Project manager 1]
		I was somewhat unfamiliar with this matter, and my involvement was largely due to my official position. As this project is only one part of my responsibilities. While our team leader and development manager were significantly involved, I have predominantly been on the listening side. [PES manager 3]
